# Reciprocity and exclusion in informal financial institutions: An experimental study of rotating savings and credit associations

**DOI:** 10.1371/journal.pone.0202878

**Published:** 2018-08-29

**Authors:** Shimpei Koike, Mayuko Nakamaru, Tokinao Otaka, Hajime Shimao, Ken-Ichi Shimomura, Takehiko Yamato

**Affiliations:** 1 Department of Value and Decision Science, Tokyo Institute of Technology, Tokyo, Japan; 2 School of Environment and Society, Tokyo Institute of Technology, Tokyo, Japan; 3 Department of Social Engineering, Tokyo Institute of Technology, Tokyo, Japan; 4 Research Institute for Economics and Business Administration, Kobe University, Kobe, Japan; 5 School of Engineering, Tokyo Institute of Technology, Tokyo, Japan; City, University of London, UNITED KINGDOM

## Abstract

Group cooperation is fundamental to human society. The public goods game is often used to describe the difficulty of group cooperation. However, there are other structures of institutions to maintain group cooperation such as Rotating savings and credit associations (ROSCAs). ROSCAs are informal financial institutions that exist worldwide, in which all participants contribute to a common fund and take turns to receive a return. ROSCAs are common in developing countries and among migrant groups in developed countries. There are various types of ROSCAs, and they share a crucial problem in that participants whose turn to receive a return has passed have an incentive to default on their contributions. We conducted a laboratory experiment to investigate the mechanisms that can prevent default in a fixed ROSCA, in which the order of receipt of returns is determined before starting and is also known to members. The findings are as follows. (i) Excluding low contributors from ROSCA groups by voting increased contribution rates both before and after the receipt of returns. (ii) ROSCA members exhibited reciprocity and a sense of revenge: that is, members contributed to the returns payments of other members who had contributed to them, and did not contribute to the returns payments of non-contributors. Voluntary behaviors thus sustained ROSCAs. Meanwhile, an exogenous punishment whereby subjects were prevented from receiving returns payments unless they had themselves contributed previously did not increase contribution rates.

## Introduction

The evolution of cooperation is one of unsolved research topics from the viewpoint of not only evolutionary theory but also social sciences such as economics, social psychology, and sociology. The recent studies show there are several mechanisms to promote not only cooperation between two players but also group cooperation (e.g. [1, 2]): kin selection [3], direct reciprocity [4, 5], indirect reciprocity [6–9], network reciprocity [10–12], group selection [13, 14]. In addition to these five mechanisms, the effect of punishment has been discussed [15–18]. Even though group cooperation has various structures in our society, many studies about group cooperation has been investigated by using the public goods game, in which all the members of the group contribute to the fund, the pool or the public goods, and receive the benefit from the public goods equally. Besides the public goods game, there are other types of games; Diekmann [19] proposed the volunteer's dilemma game in which one of the group members contributes to the goods, and all of them receive returns equally. The example of the volunteer's dilemma game is that each housemate cooks dinner or cleans the kitchen and the bathroom for members in rotation when several people share the house. Koike et al. [20] proposed the rotating indivisible goods game, in which all the group members equally contribute to the fund or the goods, and one of them receives returns in rotation. The example of the rotating indivisible goods game is reciprocal groups named rotating savings and credit associations (ROSCAs). In this article, we focus on ROSCAs to clarify the mechanism of preventing the social dilemma of the rotating indivisible goods game.

ROSCAs are informal financial institutions that exist worldwide. They are common in developing countries and among migrant groups in developed countries [21–28]. Ardener [22] defined ROSCAs as “association(s) formed upon a core of participants who agree to make regular contributions to a fund which is given, in whole or in part, to each contributor in rotation.” If *n* participants join a ROSCA, they will arrange regular meetings to be held *n* times. At every meeting, each participant contributes a fixed sum of money to a communal fund, which is then paid to one participant as their return for participation. By the end of the *n*-th meeting, all participants should have made *n* contributions and received one returns payout. ROSCAs are similar to the public goods game in that all participants equally contribute to the group, but the difference is that only one participant can receive their returns at a time in ROSCAs.

ROSCA participants typically use their returns payouts to buy durable goods [29], invest in a business [23], or make precautionary savings to meet unplanned expenses [30]. Some microfinance organizations, such as the Grameen Bank, and credit cooperatives have originated from ROSCAs [31]. The advantage of joining a ROSCA is that, with the exception of the last recipient, participants obtain access to money earlier than would have been possible through independent saving. Generally, ROSCAs can be classified into three types according to the means used to determine the order of payment of returns: a random ROSCA, a fixed ROSCA, and a bidding ROSCA. With the random ROSCA, at each meeting a recipient is selected by lottery from among those participants who have not yet received their returns. On average, participants in a random ROSCA can expect to receive their fund at the (*n*+1)/ 2-th meeting. In the case of the fixed ROSCA, the order of payment is determined before the first meeting. Finally, in the bidding ROSCA, participants bid for the available returns payout at each meeting, and payment goes to the highest bidder.

ROSCAs is one of the most common finance institute in the developing countries such as Africa and some Asian countries, which supports socioeconomic development and improves human welfare for those people [32, 33]. Ambec and Treich [34] defined the benefit of ROSCAs: facilitating an early purchase of a durable good, as a substitute to insurance, coping with self-control problems. One important characteristic of ROSCAs is that contributions are voluntary. In a successfully managed ROSCA, all participants continue to contribute after receiving their returns. Defaulters who fail to keep up their contributions after receiving their returns payouts save a sum of money equal to those missed contributions. When this occurs the other participants receive less than they would have if all had contributed as promised. Thus the question arises of what ROSCAs can do to prevent such defaults.

In this article, we focus on social connectedness among ROSCA participants, which is key to preventing defaults [35, 36]. Participants can obtain information about potential defaulters from their social connections, and participant reputation is important in the formation of ROSCA groups. Excluding unreliable participants decreases the risk of default, and the threat of exclusion deters group members from defaulting. Koike et al. [20] conducted evolutionary game simulations to investigate the effect on sustaining ROSCAs of excluding unreliable ROSCA members based on their reputation—a process termed peer selection. Besides peer selection, the forfeiture rule is applied, which prevents a non-contributing member from receiving any payout. In a mutual-aid game, which closely resembles a ROSCA, the forfeiture rule can be interpreted as a costless punishment [37]. Koike et al. [20] showed that the combination of peer selection and the forfeiture rule can prevent defaulters and maintain ROSCAs.

In the present study, we conducted a laboratory experiment to examine whether these two factors prevented subjects from becoming defaulters and sustained fixed ROSCAs, where participants are shuffled randomly for each cycle, and the order of payment is determined before each cycle starts. During each cycle, participants decide to contribute the fund or not, and the fund is given to the predetermined receiver in each meeting. Our participants were undergraduate and graduate students who were not familiar with ROSCAs. Our experiment showed that peer selection through voting increases contribution rates both before and after participants receive their payouts by excluding low contributors and allowing participation by medium and high contributors. Accordingly, the mean payoff for subjects who participated in the fixed ROSCA with peer selection significantly exceeded that for those who participated in the fixed ROSCA without peer selection. However, the forfeiture rule did not increase contribution rates because almost all subjects responded to a defaulter by refusing to contribute to the fund destined for that defaulter. Therefore, our results suggest that the use of reputation to exclude defaulters can solve the default problem affecting ROSCAs even without external enforcement through a mechanism like the forfeiture rule.

This paper proceeds as follows. In the next section, we review the related literature. The third section describes the design of our experiment and theoretical predictions based on evolutionary game simulations. The fourth and fifth sections then present our results and conclusions, respectively.

### Related literature

Several theoretical studies have investigated the conditions for solving the default problem and sustaining ROSCAs. Besley et al. [38] showed that the random ROSCA is maintained if the benefit to the first recipient who defaults does not exceed the default cost. The default cost represents social sanctions: defaulters gain a bad reputation, are excluded from future ROSCAs, or suffer damage to personal property. Anderson et al. [39] investigated random and fixed ROSCAs, under conditions where the same individuals repeatedly became members. The authors showed that the first recipient is always inclined to leave and chooses defection, despite the cost of exclusion from all future meetings. Their field survey in Kenya reported that ROSCA members enforced social punishments, such as confiscating the property of defaulters. However, the argument of Anderson et al. [39] depends on the assumption that the same members repeatedly join the same ROSCA: they did not investigate whether exclusion can prevent defaulters in a situation where ROSCA membership is changing.

To the best of our knowledge, no experimental study of ROSCAs has been undertaken. Using public goods experiments, several studies have found that exclusion based on reputation can promote contribution level by expelling low contributors from the game [40–43]. This exclusion can be interpreted as a costless punishment. In contrast, a number of experimental studies have investigated costly punishments. Costly punishment of free riders has been found to increase contribution levels in public goods experiments [44–50]. However, Herrmann et al. [51] suggested that antisocial punishment, in which free riders pay a cost to punish contributors, is observed in societies with weak civic cooperation and rule of law. Under some social conditions, costly punishment is ineffective in increasing contribution level [52–54].

A ROSCA resembles both the simultaneous and sequential public goods games, although each is structurally unique. In joining a ROSCA, all players decide whether to make a series of simultaneous contributions of a fixed sum to a fund while each taking turns to receive a return. In the public goods games, players decide their contribution level to a public good, either simultaneously or sequentially, and all benefit from the public good. Additionally, the payoff function of a player in our ROSCA game depends on the contributions of others rather than themselves. Conversely, the payoff function of a player in the public goods games is determined by the contributions of all players, including themselves.

## Experiment

### Design

The Tokyo Institute of Technology Ethics Committee approved our study on November 19, 2012, and October 10, 2014, and two approval numbers are 2012030 and 2014041, respectively. All subjects provided their written informed consent to participate in the experiment. The experiment consisted of four types of sessions: Treatment B (Base); Treatment P (Punishment); Treatment V (Voting); and Treatment VP (Voting with Punishment). In Treatment P, subjects played the fixed ROSCA on condition that non-contributing members could not receive return payouts (punishment rule). In Treatment V, subjects mutually select group members by voting. In Treatment VP, both the voting and punishment rules were employed. In Treatment B, neither the voting rule nor punishment rule was used.

#### Treatment B (Base)

For each treatment, each session consisted of ten rounds, each comprising four periods. At the beginning of a session, 20 subjects were each assigned to a laboratory computer and given instructions (see S1−S4 Instruction). After listening to the experimenter read aloud the instructions, the subjects were randomly divided into five groups, each comprising four members. We informed the subjects up-front that they would play the four periods at each round. At the beginning of each round, an order of payment was randomly determined and displayed on subjects’ computer screens, such that each group member knew the order in which all members of their group would receive their payouts. Notice that the number of periods is equal to the number of members and that each member could receive her payout exactly one time. The member scheduled to receive their payout in the *n-*th period was termed the *n-*th recipient.

In each period, the experimenter gave 100 points to all group members, and all members other than the scheduled recipient had to choose whether to contribute these points to the communal fund or retain them. Members that decided to contribute lost 100 points, while members that decided not to contribute saved 100 points. We assumed that members who received their returns payouts earlier would profit more than those who received them later. This was because the earlier members received their payouts, the earlier they could purchase durable goods, invest in their business and make a profit. If *m*_C_ is the number of contributing members and *n* is the number of periods, the real payoff to each recipient is

$$\pi_{m_{C,}n}=100(\frac{m_{C}}{{\left(1+\delta \right)}^{n-4}}+1),$$

where *δ* is the discount factor. We used *δ* = 0.3 in this experiment.

We informed the subjects up-front that their payoffs are determined by the above equation and that the discount factorδis equal to 0.3. We also provided each subject a table showing how his/her payoff in one round consisting of four periods is determined by both “the order of payout receipt” and “the number of other members of his/her group who give 100 points to the fund” with this discount factor (Table 1).

**Table 1:** Payoffs for receiving returns payouts in each period in *δ* = 0.3.

Period	Number of contributors to the fund			
	0	1	2	3
**First**	100	320	539	759
**Second**	100	269	438	607
**Third**	100	230	360	490
**Fourth**	100	200	300	400

**Fig 1:**
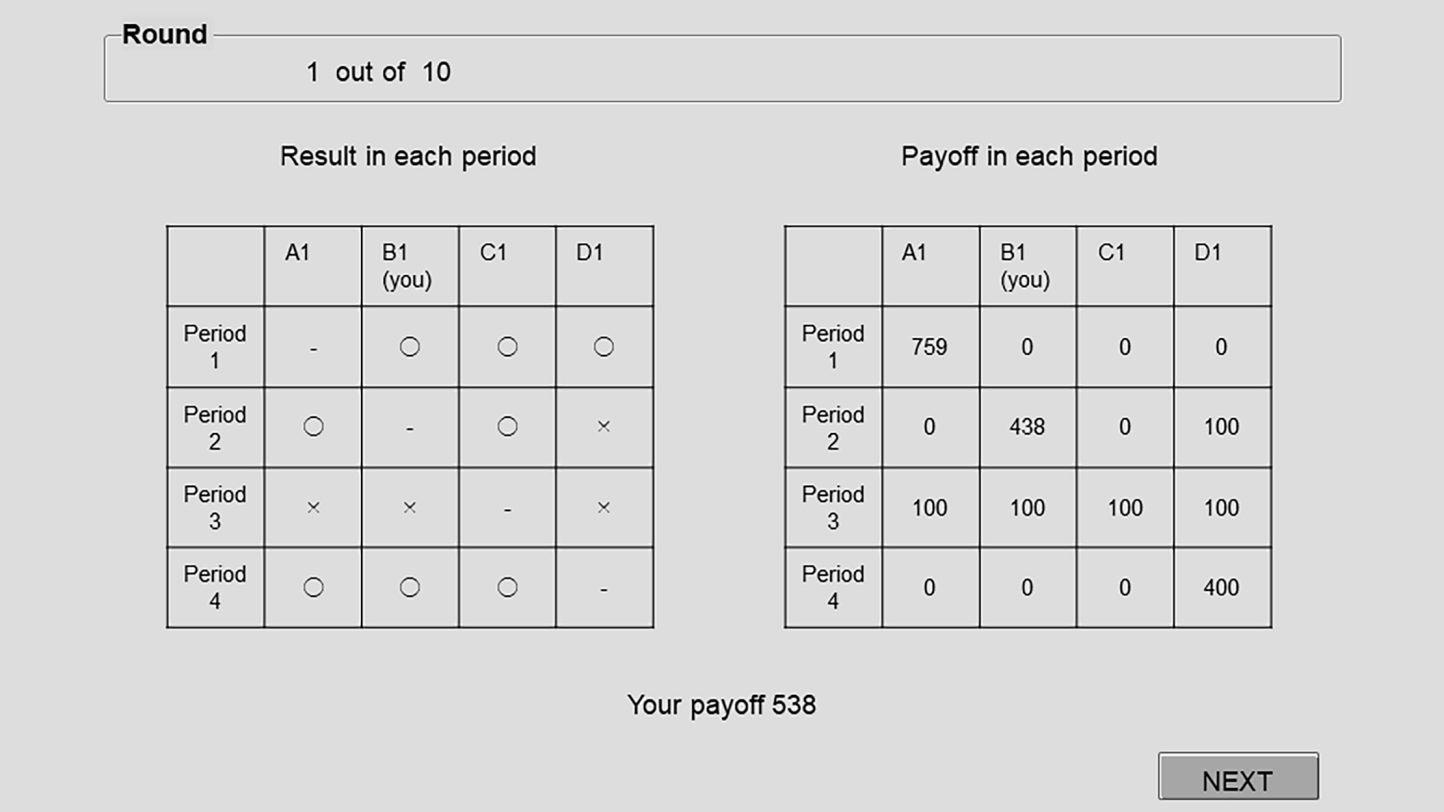
Example of a confirmation screen. “O” means choosing “GIVE”, “x” means choosing “NOT GIVE”, and “-” indicates the scheduled recipient for the relevant period.

After the receipt of each returns payout, all members were informed which members had contributed to the fund and which had received a returns payout (Fig 1).

**Fig 2:**
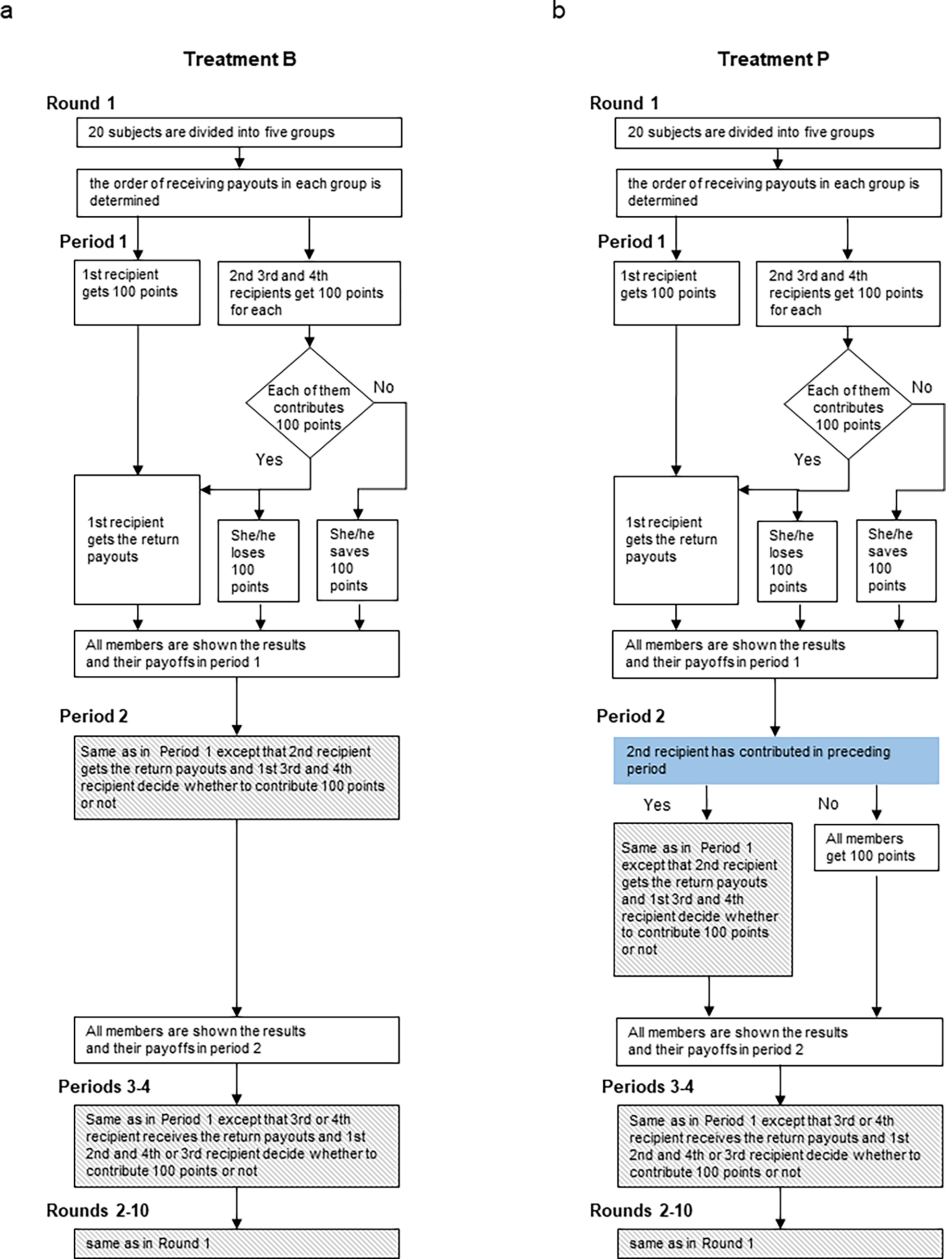
Experimental procedures for Treatments B and P.

Fig 2 (Panel a) summarizes the experimental procedure for Treatment B. Let us describe how the experiment proceeds and payoffs are determined in Treatment B by using the following example illustrated in Fig 1. This example was explained to the subjects in the experimental instructions.

### Example 1

Consider the group of four members {A1, B1, C1, D1}. Now suppose that the order of payout receipt is “A1 → B1 → C1 → D1”. In the first period, A1 receives the payout. Each of the three members, B1, C1, and D1, chooses either to “GIVE” 100 points to A1 or to “Not Give” any point (0 point) to A1. Suppose that all three members except A1 choose “GIVE” (the mark “O” in Fig 1). Then A1 receives 300 points and his or her payoff will be 759 at the end of the fourth period: 300×1.3(4−1) + 100 ≒ 759. The payoffs of the other three members are 0.

In the second period, B1 receives the payout. Suppose that A1 and C1 choose “GIVE”, and D1 chooses “NOT GIVE” (the mark “x” in Fig 1). Then B1 receives 200 points and his/her payoff will be 438 at the end of the fourth period: 200×1.3^(4–2)^ + 100 = 438. The payoffs of A1 and C1 are 0 and the payoff of D1 is 100.

In the third period, C1 receives the payout. Suppose that all three members except C1 choose “NOT GIVE”. The payoffs of all four members are 100.

In the fourth period, D1 receives the payout. Suppose that all three members except D1 choose “GIVE”. Then D1 receives 300 points and his or her payoff is 400 at the end of the fourth period: 300×1.3(4−4) +100 = 400. The payoffs of the other three members are 0. The total payoff of B1 (you) in this round is 0 + 438 + 100 + 0 = 538, as appeared at the bottom of the screen in Fig 1. After the fourth period, each member was shown his/her total payoff in that round.

At the beginning of the next round, new groups were randomly formed, with members being unaware of whether their new group still contained any members from their group in the previous round. After the end of the final round, the subjects answered a questionnaire and were paid a reward proportional their total payoff for the session.

### Treatment P (Punishment)

We introduced a rule called the punishment rule: this rule holds that no member who had not contributed in preceding periods could receive a returns payout. In Example 1 for Treatment B, because the scheduled fourth recipient, D1, did not contribute in the second period, D1 cannot receive the payout in the fourth period, but save 100 points in Treatment P. In this situation the other members also save 100 points because they do not need to contribute 100 points. Koike et al. [20] introduce this rule and they term it the forfeiture rule. Notice that this punishment rule is applied to a member who has not contributed BEFORE receiving his/her payout. There is no penalty for a member who has stopped contributing AFTER receiving his/her payout, such as A1 in Example 1. Fig 2 (Panel b) summarizes the experimental procedure for Treatment P.

### Treatments V (Voting) and VP (Voting with Punishment)

**Fig 3:**
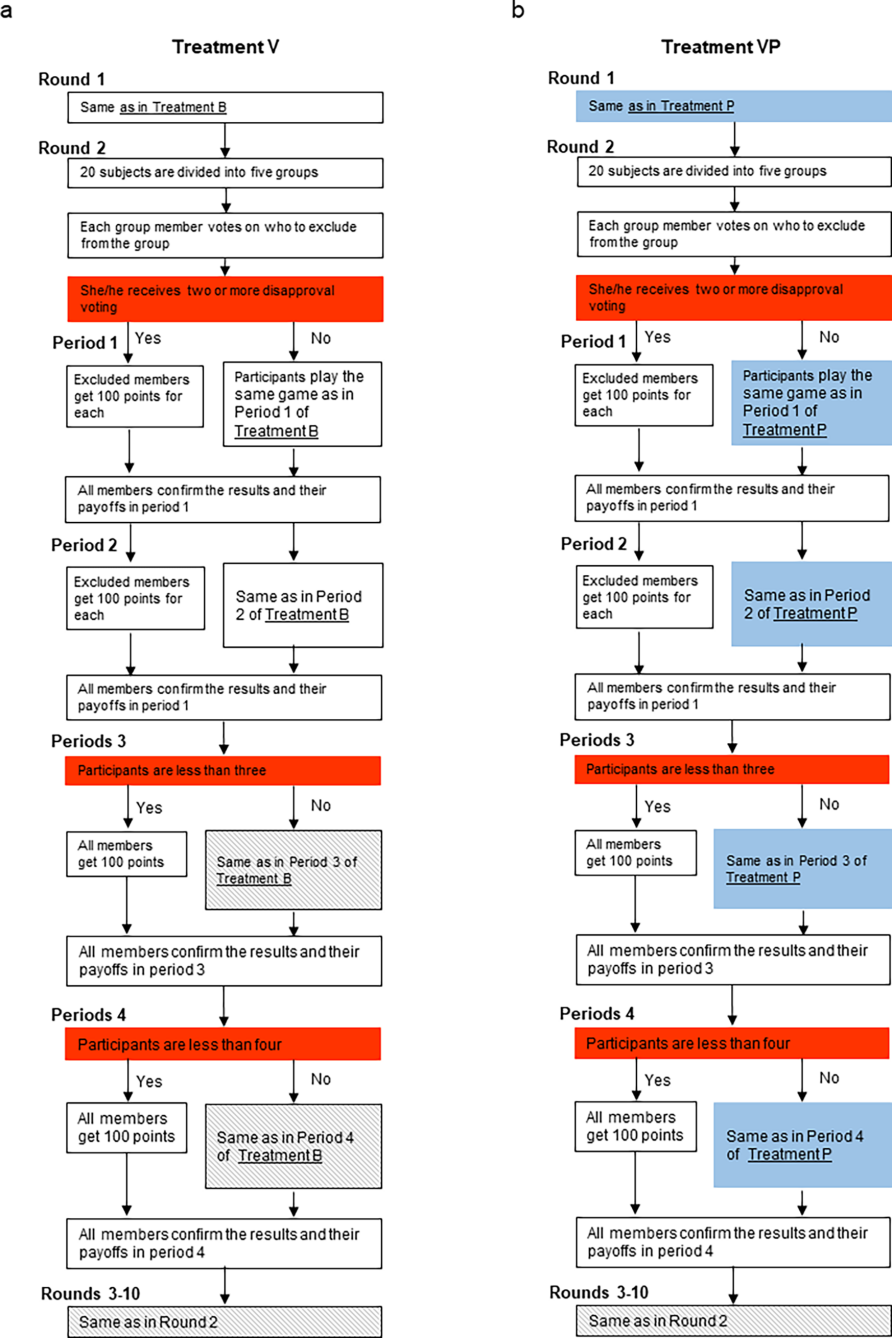
Experimental procedures for Treatments V and VP.

We introduced a voting system. In this system, 20 subjects were randomly divided into five groups at the beginning of each round. All group members were aware of both the number of times each member had contributed in previous rounds and the number of previous rounds. Based on this information, starting in the second round each member voted on who to exclude from the group. A group member was expelled if at least two members voted to exclude them. Excluded members were paid 100 points in each period and saved a total of 400 points during one round but could not participate in the round in which they were expelled. At the end of each round, the 20 subjects were randomly divided into five groups for the next round. Therefore, subjects excluded from their group in a previous round had the chance to join a new group in the next round. Treatment VP was the same as Treatment P with the addition of the voting system. Fig 3 summarizes the experimental procedures for Treatments V and VP.

We conducted two sessions for each treatment at the Tokyo Institute of Technology from December 2011 to June 2013 using the program z-Tree (Fischbacher 2007). We recruited 160 (20 × 4 × 2) subjects from a Tokyo Institute of Technology subject pool. Subjects consisted mainly of undergraduate students who had never participated in similar experiments. The average payment made to the subjects was 3,506 yen (approximately 35.06 US dollars at a rate of 1 US dollar = 100 yen).

### Theoretical predictions

We analyze four-person four-period ROSCA games in the four treatments, and obtain the subgame-perfect equilibrium, whereby no player pays a contribution during any period in any of the treatments (the proof appears in S1 Appendix).

Applying backward induction to the ROSCA game with Treatment B, the fourth scheduled recipient gets nothing in the fourth period, because the other players have no incentive to contribute at the end of the round. The fourth recipient has no incentive to contribute in the preceding periods and saves 100 points in each period on his own. Therefore, the third scheduled recipient also gets nothing in the third period because he has not received a contribution from the first and second recipients, who have already received their returns payouts, or from the fourth scheduled recipient. Similarly, the other recipients get nothing in the period when they receive the returns payout and have no incentive to contribute before or after receiving that payout.

We also consider what happens in the ROSCA game with Treatment P. In this treatment the other players also have no incentive to contribute to the fund in the fourth period and then the fourth scheduled recipient receives zero. If the fourth recipient cannot receive their payout because of the punishment rule for non-contribution before receiving the fund, he chooses no-contribution in the preceding periods and so independently saves 100 points in each period. As with the game in Treatment B, the third scheduled recipient also gets nothing in the third period because he has received no contribution from the other recipients. Hence, in Treatment P, no players pay any contribution during rounds 1–10.

The theoretical prediction in Treatment V is as follows. In round 10, the players allowed to participate in a group pay no contribution to save 100 points each, regardless of history. Meanwhile, the excluded members also save 100 points per period. At the beginning of the round, each player knows that all participants will receive the same points and his vote does not affect his payoff. Applying backward induction, this argument is true not only for round 10 but also for rounds 2–9; hence, in rounds 2–10, all players elected as members of groups pay no contribution in any period. As the argument for round 1 is identical to that for Treatment B, in round 1, no player pays any contribution in any period.

Treatment VP is a combination of Treatments P and V. Therefore, no player pays any contribution at any period in rounds 1–10, and no player cares about voting in rounds 2–10.

## Results

### Average contribution rates over time and distribution of total profit

We categorize the contribution rates into two types: the contribution rate before receiving the payout (*β*_0_) and the contribution rate after receiving the payout (*α*_0_). We consider Table 2 to understand *β*_0_ and *α*_0_. Because A1 is the first recipient, *β*_0_ cannot be calculated, but *α*_0_ is 1/3 because he has made a contribution once out of a possible three times after receiving the funds. Likewise, the *β*_0_ and *α*_0_ of B1 are 1/1 (= 1) and 2/2 (= 1), and those of C1 are 2/2 and 0/1, respectively. The *β*_0_ of D1 is 2/3, but *α*_0_ cannot be calculated because he is the last recipient.

**Table 2:** Example of calculating *β*_0_, *α*_0_, *α*_1_, *α*_2_ and *α*_3_.

		Subject			
		A1	B1	C1	D1
**Period**	First	-	o	o	x
	Second	o	-	o	o
	Third	x	o	-	o
	Fourth	x	o	x	-
***β*_0_**		-	1/1	2/2	2/3
***α*_0_**		1/3	2/2	0/1	-
***α*_1_**		1/2	1/1	-	-
***α*_2_**		-	1/1	0/1	-
***α*_3_**		0/1	-	-	-

*Notes*: “o” means choosing “GIVE”, “x” means choosing “NOT GIVE”, and “-” indicates the scheduled recipient in that period.

**Result 1.** The average contribution rate before receiving the payout in Treatment V was significantly higher than in Treatments B, P, or VP. The average contribution rate before receiving the payout in Treatment B was not significantly different from that in Treatment P.

**Fig 4:**
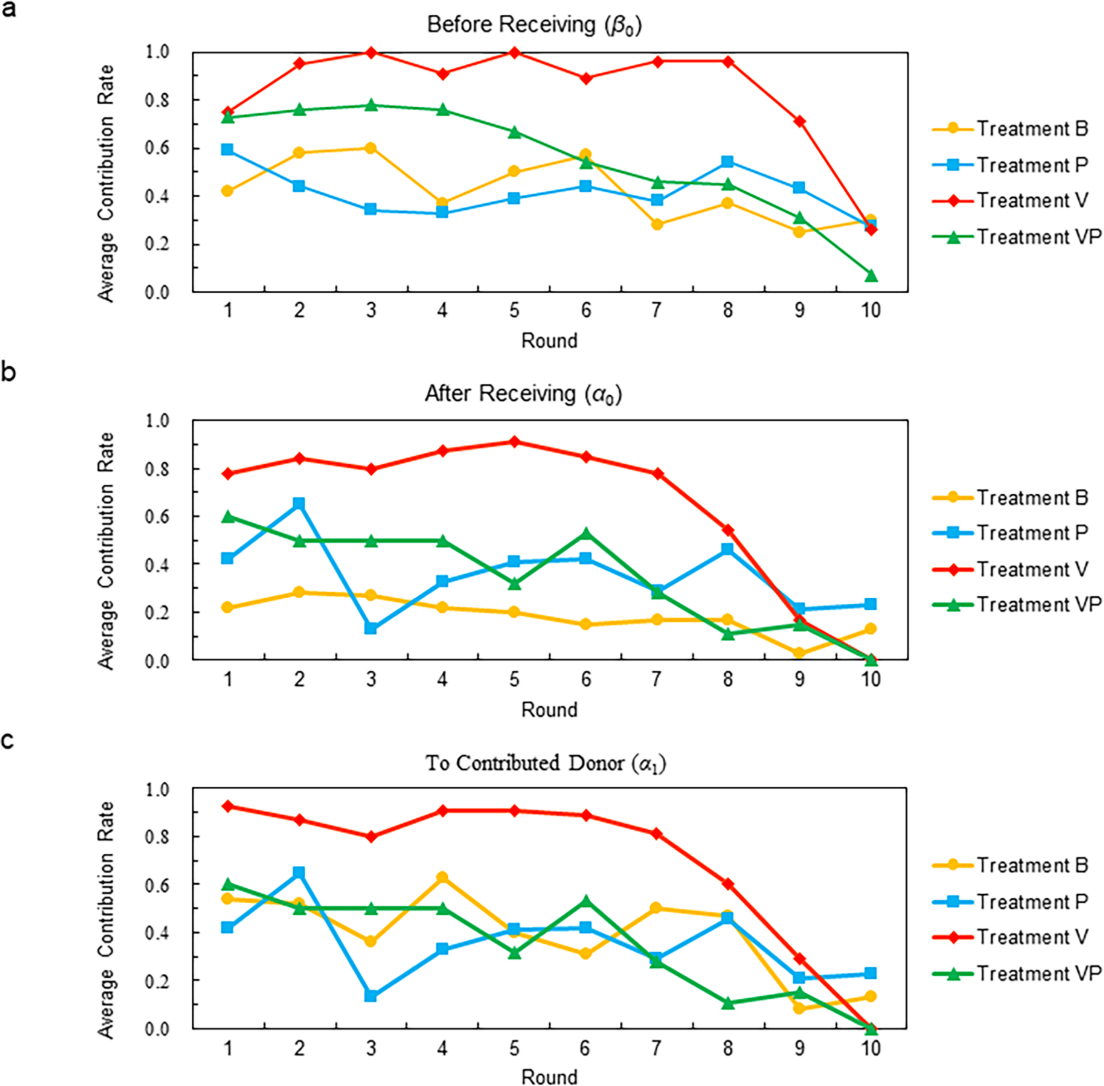
Average contribution rates in each round.

**Support.** Fig 4 (Panel a) shows the average *β*_0_ per subject in each round. We conducted a Steel–Dwass rank sum test to compare the four treatments. The average *β*_0_ per subject in Treatment V (red line) was significantly higher than the rates in Treatment B (yellow line) except in the final round, and was significantly higher than the rates in Treatment P (blue line) in eight of ten rounds and in Treatment VP (green line) in five of ten rounds (*p* < 0.05). However, except in round 3, no significant difference existed between the average *β*_0_ per subject in Treatments B and P. The average values of *β*_0_ in Treatment VP did not significantly differ from those in Treatment B in nine of ten rounds and did not differ from those in Treatment P in eight of ten rounds. In the final round, the end-game effect caused the average values of *β*_0_ in Treatments VP and V to decrease. This tendency is the same as in results observed in public goods experiments with majority voting (Cinyabuguma, Page, and Putterman 2005; Maier-Rigaud, Martinsson, and Staffiero 2010; Croson, Fatas, Neugebauer, and Morales 2015).

**Result 2.** The average contribution rate after receiving the payout in Treatment V was significantly higher than in Treatments B, P, or VP. The average contribution rates after receiving the payout do not differ significantly among Treatments B, P, and VP.

**Support.** Fig 4 (Panel b) shows the average *α*_0_ per subject in each round. The average *α*_0_ per subject in Treatment V was significantly higher than in Treatment B, except in rounds 9 and 10 (*p* < 0.05). To canvass the results of the treatments with and without the punishment rule, we categorized the contribution rate after receiving the returns payout (*α*_0_) into three types: the contribution rate of a subject to a recipient who has contributed to both the subject and all other members (*α*_1_); the contribution rate of a subject to a recipient who has contributed to the subject but not to some other members (*α*_2_); and the contribution rate of a subject to a recipient who has not contributed to the subject (*α*_3_). We reconsider Table 2 to confirm these values. In Table 2, *α*_1_ of subject A1 is 1/2 because subjects B1 and C1 contribute to A1 in period 1, while A1 contributes to B2 in period 2 and does not contribute to C1 in period 3. Moreover, the *α*_2_ of B1 is 1/1 because B1 contributes to D1, who contributes to both B1 and C1 but does not contribute to A1 in period 1. Moreover, the *α*_3_ of A1 is 0/1, because D1 does not contribute to A1 in period 1 and A1 does not contribute to D1 in period 4.

The punishment rule prohibited subjects from contributing to any scheduled recipient who had previously not contributed themselves, and so *α*_0_ in Treatments P and VP was represented by *α*_1_. Moreover, *α*_0_ in Treatments B and V was represented by the contribution rate of a subject to the recipient regardless of that recipient’s past behavior. Hereafter, we use *α*_1_ rather than *α*_0_ in comparing the contribution rate after receipt of the funds in the four treatments under the same criteria.

Fig 4 (Panel c) shows the average value of *α*_1_ per subject in each round. In Treatment V, the average *α*_1_ per subject was significantly higher than in Treatment P in six of ten rounds, while in Treatment VP it was higher in seven of ten rounds (*p* < 0.05). However, the average *α*_1_ per subject did not differ significantly among Treatments B, P, and VP in any round (*p* > 0.05).

**Result 3.** The mean payoff in Treatment V significantly exceeded that in Treatments B, P, and VP. The mean payoffs in Treatments B, P, and VP were not significantly different.

**Fig 5:**
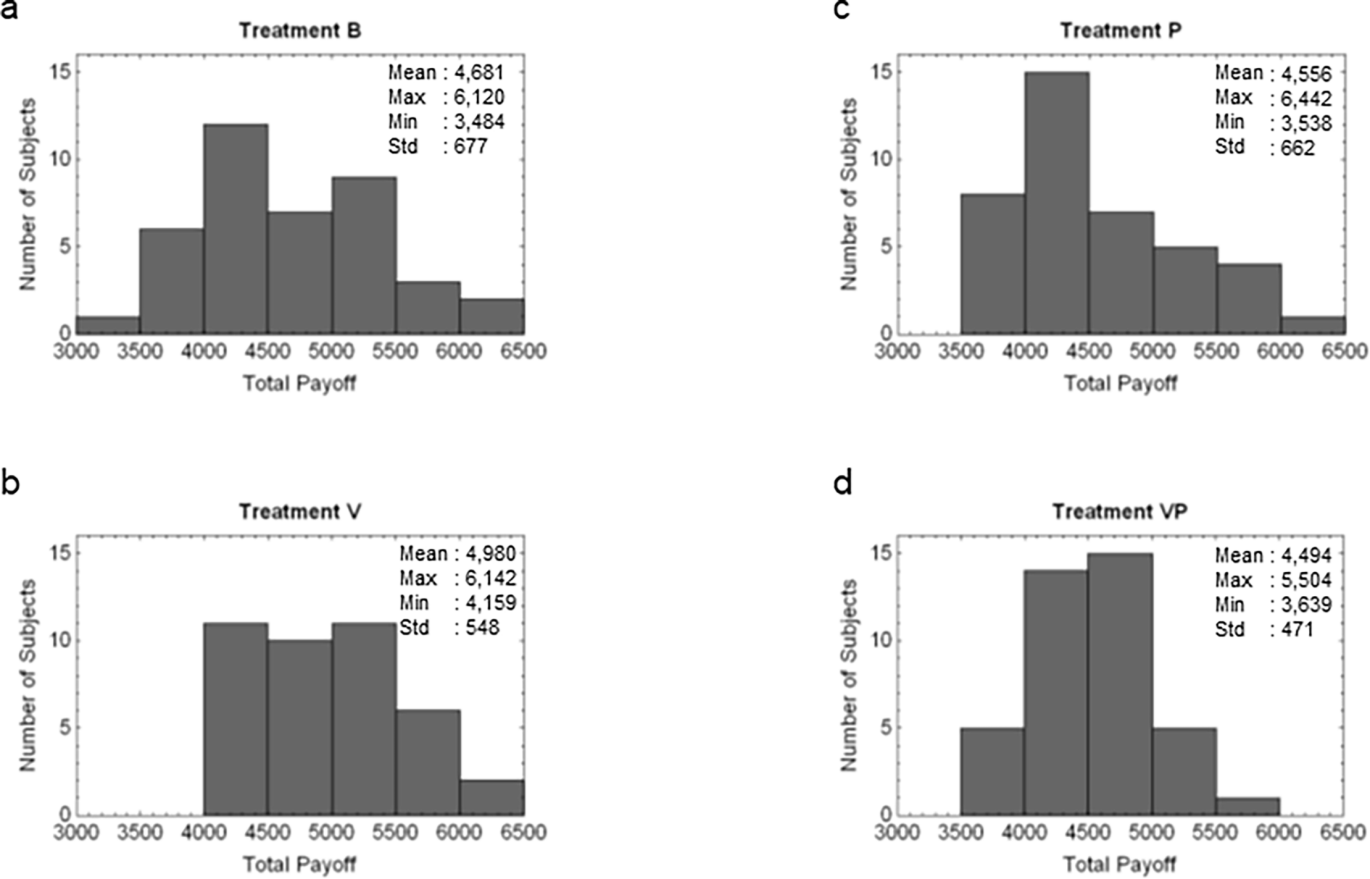
Total profits after the treatments.

**Support.** We now consider the distribution of the total payoff at the end of the treatments (Fig 5). Fig 5 (Panel b) shows that the payoffs for all subjects in Treatment V exceeded 4,000 points, which equaled the amount that all subjects received during one treatment (100 points × 4 times × 10 rounds). The payoffs for seven, eight, and five subjects were below 4,000 points in Treatments B, P, and VP, respectively. The mean payoff in Treatment V was 4,980 points, significantly higher than the mean payoffs in Treatments B, P, and VP, which were 4,681, 4,566, and 4,494, respectively (*p* < 0.01 in Wilcoxon rank-sum test). The mean payoffs thus were not significantly different (*p* > 0.10).

### Why did the voting system work well?

**Fig 6:**
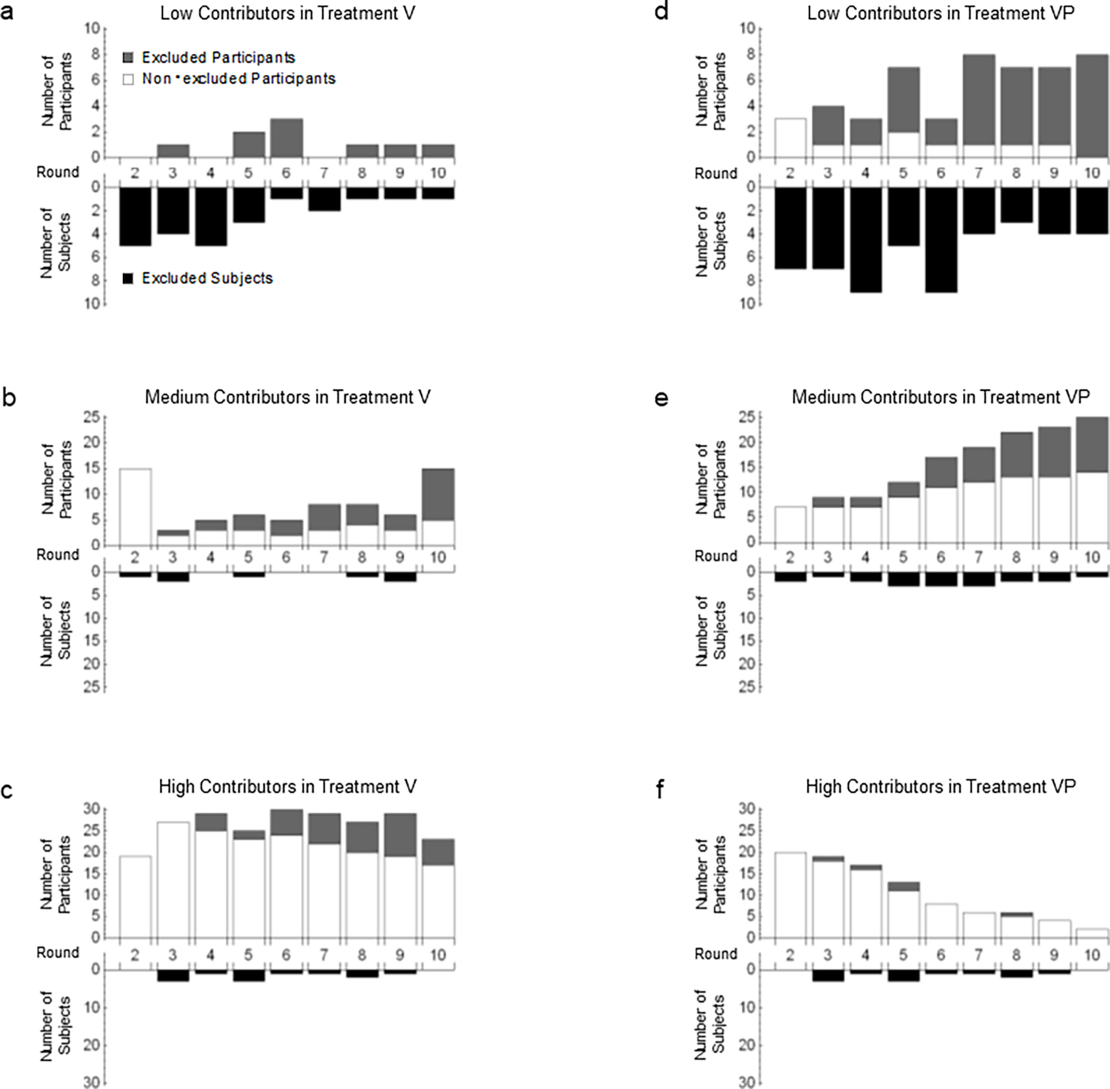
Total numbers of participants and excluded subjects in each round over two sessions.

To show why the voting system boosted contribution rates, we investigated both the number of participants in each group and the number of subjects excluded from each group. In Fig 6, the subjects are categorized into three types based on cumulative contribution rate: low contributors, with a cumulative contribution rate of less than 0.40; medium contributors, with a cumulative contribution rate of 0.40–0.80; and high contributors, with a cumulative contribution rate equal to or greater than 0.80. The upper sections in each part of Fig 6 show the number of participants who were never excluded from the groups (non-excluded participants are represented by white bars) and the number who were excluded in previous rounds (excluded participants are represented by gray bars). The lower section in each part of Fig 6 indicates the number of subjects who were excluded from the groups (excluded subjects are represented by black bars). The horizontal axis represents the number of rounds; the upper and lower vertical axes represent the numbers of participants and subjects, respectively.

**Result 4.** The voting system excluded low contributors while letting medium and high contributors participate in Treatments V and VP.

**Support.** In Treatment V, low contributors were frequently excluded from the groups (Fig 6 Panel a); medium and high contributors were less frequently excluded (Fig 5 Panel b and Panelc). Over the 10 rounds in Treatment V, the voting system excluded 72% of low contributors and 6% of medium and high contributors. The difference was significant (*p* < 0.01 by Fisher’s exact test). Therefore, because the voting system excluded low contributors from the groups while favoring participation by medium and high contributors, the contribution rates in Treatment V exceeded those in Treatments B and P, which lacked the voting system.

In Treatment VP, the number of excluded subjects did not always exceed the number of participants in low contributors (Fig 6 Panel d); however, among medium and high contributors, excluded subjects were outnumbered by actual participants in each round (Fig 6 Panel e and Panel f). Because the average contribution rates (*β*_0_, *α*_1_) continued declining with number of rounds after this number reached the halfway point (Fig 4), the number of high contributors decreased while that of medium contributors increased. Over the 10 rounds in Treatment VP, the voting system excluded 51% of low contributors versus only 8% of medium and high contributors. The exclusion rates thus differed significantly among the three groups (*p* < 0.01).

**Result 5.** The average exclusion rate of low contributors in Treatment V significantly exceeded that in Treatment VP.

**Support.** The average exclusion rate of low contributors in Treatment V (72%) was significantly higher than in Treatment VP (51%, *p* = 0.043).

While, in a comparison of Treatments V and VP, the exclusion rates of medium and high contributors were 6% and 8%, respectively, and thus were not significantly different (*p* = 0.405). In sum, the contribution rates in Treatment V exceeded those in Treatment VP.

**Result 6.** The voting system gave excluded low contributors the chance to participate in future groups in Treatments V and VP.

**Fig 7:**
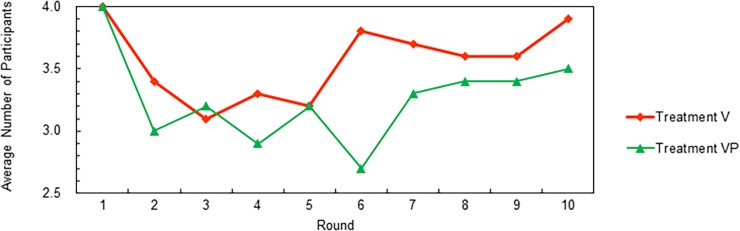
Average number of participants in each round.

**Support.** The upper part of Fig 6 (Panel a) shows that all the low contributors available to participate were excluded. In Treatment VP, similarly, 39 out of 50 low contributors available to participate were excluded (Fig 6 Panel b). As low contributors who had been excluded had a chance to participate in a group again, the number of participants included in each round in Treatment V averaged at least three out of every four participants available for selection (Fig 7).

### Why did the punishment rule not work well?

The previous sections showed that the contribution rates did not differ significantly between Treatments B and P, and that the contribution rates in Treatment V exceeded those in Treatment VP. These results suggest that punishment did not work well. To examine the reason, we focus on the differences among the average values of *α*_1_, *α*_2_, and *α*_3_ per subject over ten rounds.

**Result 7.** The average contribution rate to non-contributors was significantly lower than that to contributors in Treatments B and V without the punishment rule.

**Support.** Table 3 shows that the average values of *α*_1_, *α*_2_, and *α*_3_ in Treatment B were 0.40, 0.33, and 0.04, respectively. Thus the average values of *α*_1_ and *α*_2_ were not significantly different (*p* = 0.59 by the Steel–Dwass rank sum test), while that of *α*_3_ was significantly smaller than the other two (*p* < 0.01). This suggests that the subjects in Treatment B discriminated between recipients based on whether those recipients had contributed to them, and rejected making contributions themselves to non-contributors. This behavior can be interpreted as a kind of costless punishment of non-contributors. Hence, subjects tended to punish recipients who had not contributed to them in Treatment B. As a result, even though Treatment P combines Treatment B with the punishment rule that prohibited subjects from contributing to a recipient who had not previously contributed, the contribution rates in Treatments B and P did not differ significantly.

**Table 3:** Average contribution rates in the four treatments over 10 rounds.

	Before receiving	After receiving			
	*β*_0_	*α*_0_	*α*_1_	*α*_2_	*α*_3_
**Treatment B**	0.44	0.18	0.40	0.33	0.04
	(0.05)	(0.03)	(0.05)	(0.08)	(0.02)
*N*	40	40	39	30	40
**Treatment P**	0.40	0.29	0.29	-	-
	(0.05)	(0.06)	(0.06)		
*N*	40	38	38		
**Treatment V**	0.81	0.62	0.71	0.60	0.15
	(0.03)	(0.03)	(0.04)	(0.09)	(0.04)
*N*	40	40	40	28	37
**Treatment VP**	0.56	0.38	0.38	-	-
	(0.04)	(0.05)	(0.05)		
*N*	40	39	39		

Averages with standard errors in parenthesis.

Similarly, in Treatment V, the average values of *α*_1_, *α*_2_, and *α*_3_ were 0.71, 0.60, and 0.15, respectively (Table 3). The average values of *α*_1_ and *α*_2_ did not differ significantly (*p* = 0.99), while the average value of *α*_3_ was significantly smaller than the others (*p* < 0.01). This suggests that the subjects in Treatment V also retaliated against those who had not contributed to them.

### Order effect for the receipt of payouts

In our experiment, each subject was informed of the order in which they would receive the returns payout in each round. Here, we examine how this order influenced contribution rates.

**Result 8.** The average contribution rates before recipients received their returns payouts gradually decreased from the second recipient, to the third, and finally the fourth in all four treatments.

**Support.** In Table 4, the average value of *β*_0_ of the second recipients significantly exceeded that of the third recipients in all treatments (*p* < 0.05 by the Steel–Dwass rank sum test). The average value of *β*_0_ of the third recipients significantly exceeded that of the fourth recipients for all treatments (*p* < 0.05, but *p* = 0.066 in Treatment P). Focusing on the discount factor (*δ* = 0.3), we explain this phenomenon as follows. Suppose that the second recipient contributes to the first recipient. If the first recipient is a directly reciprocal player, he contributes to the returns payout in the second period. The payoff lost by the second recipient in the first period is less than the payoff for receiving the returns payout because the total contribution of the returns payout is multiplied by (1+*δ*)^2^. Hence, the second recipient has an incentive to make a contribution before receiving the returns payout. However, if the other players behave reciprocally, the payoff that the fourth recipient lost before receiving their own returns payout, which is 400 points, equals the payoff from receiving that payout given that the total contribution of the payout is multiplied by (1+*δ*) ^0^ = 1. The last recipient has no incentive to contribute to the other players, even where direct reciprocity is in effect.

**Table 4:** Order effect of contribution rates.

	Order of payout receipt			
	First	Second	Third	Fourth
**Contribution rate before payout receipt (*β***_**0**_**)**				
**Treatment B**	-	0.77	0.52	0.25
		(0.04)	(0.04)	(0.04)
*N*		100	100	100
**Treatment P**	-	0.69	0.38	0.24
		(0.05)	(0.05)	(0.04)
*N*		100	100	100
**Treatment V**	-	0.95	0.85	0.73
		(0.02)	(0.03)	(0.05)
*N*		98	94	66
**Treatment VP**	-	0.78	0.56	0.33
		(0.04)	(0.05)	(0.06)
*N*		97	81	51
**Contribution rate after payout receipt (*α***_**1**_**)**				
**Treatment B**	0.39	0.44	0.38	-
	(0.05)	(0.07)	(0.14)	
*N*	85	43	13	
**Treatment P**	0.32	0.38	0.45	-
	(0.05)	(0.07)	(0.11)	
*N*	74	43	22	
**Treatment V**	0.71	0.71	0.74	-
	(0.04)	(0.05)	(0.07)	
*N*	96	75	38	
**Treatment VP**	0.38	0.38	0.36	-
	(0.05)	(0.07)	(0.13)	
*N*	78	48	14	

Averages with standard errors in parenthesis.

**Result 9.** The average contribution rates after receiving the returns payouts among the first, second, and third recipients did not differ significantly among all four treatments.

**Support.** The average values of *α*_1_ for the first, second, and third recipients did not differ significantly among the four treatments (Table 4). We expected that the average value of *α*_1_ of the earlier recipients would be lower than that of the later recipients in Treatments B and P because earlier recipients would stop making contributions after receiving their payouts. However, because the subjects tended to behave reciprocally, the order of payout receipt did not change the average values of *α*_1_ among the first, second, and third recipients.

### Panel regression of average contribution rates

We conduct panel regression analysis to confirm the results in the previous sections. Table 5 shows the results of random and fixed effects panel regressions using *β*_0_ and *α*_1_ as dependent variables (using Breusch–Pagan Lagrange multiplier tests, *p* = 0.000). The independent variables are as follows: “Voting” and “Punishment” are dummy variables for the voting system (1 = if the subject is in Treatment V and VP) and the punishment rule (1 = if the subject is in Treatment P and VP). “Voting × Punishment” is the interaction term between “Voting” and “Punishment”. “Order” is the order of payout receipt, and subjects who receive a payout in the first period are assigned the value 1. “No. Contributor” is the number of members who contributed to the returns payout received by a subject. “No. Member” is the number of members participating in the same group as a subject. “Excluded” is a dummy for a subject having been excluded (= 1).

**Table 5:** Linear panel regressions of average contribution rates.

	Before payout receipt (*β*_0_)	After payout receipt (*α*_1_)	
	Random effects	Random effects	Fixed effects
**Voting**	0.360***	0.229***	-
	(0.058)	(0.074)	
**Punishment**	−0.078	−0.060	-
	(0.056)	(0.071)	
**Voting × Punishment**	−0.171**	−0.155	-
	(0.081)	(0.099)	
**No. Contributor**	-	0.155***	0.146***
		(0.023)	(0.024)
**No. Member**	0.084***	0.083*	0.108**
	(0.031)	(0.044)	(0.047)
**Excluded**	0.116**	0.109*	0.083
	(0.048)	(0.064)	(0.087)
**Order**	−0.213***	0.004	−0.016
	(0.014)	(0.022)	(0.024)
**Intercept**	0.702***	−0.300	-
	(0.140)	(0.187)	
**Observations**	1087	629	629
**F-test**	50.594***	12.817***	14.214***
**Adj. R**^**2**^	0.219	0.127	0.108

Robust standard errors in parentheses.

*significant at 10%

** significant at 5%

*** significant at 1%.

A panel data analysis on *β*_0_ using the Hausman test supports the random effects model (*p* = 0.401). The result of the model in the second column of Table 5 shows that the voting rule had a positive and highly significant effect on the contribution rate before receipt of payout (*β*_0_) and that the punishment rule had a negative but insignificant effect on *β*_0_. However, “Voting × Punishment” significantly and negatively affected *β*_0_. These results support Result 1. A weak positive correlation was observed between “No. Member” and *β*_0_. “Excluded” and *β*_0_ had a positive and significant correlation, which suggests that the experience of exclusion from the previous group promoted the contribution rate of excluded subjects. As mentioned in Result 8, the order of payout receipt negatively and significantly affected *β*_0_.

The Hausman test rejects the null hypotheses of zero correlation between the error term and the independent variables in the random effects model regression on *α*_1_ only at the ten percent significance level but not at the five percent level (*p* = 0.069). Therefore, we conduct the random and fixed effects models. The result of the random effects model in the third column of Table 5 shows that the voting rule positively and significantly affected the contribution rate after receipt of payout (*α*_1_) and that the punishment rule and “Voting × Punishment” had a negative but insignificant effect on *α*_1_. These results support Result 2. The results of the random and fixed effects models show that “No. Contributors” positively and significantly affected *α*_1_. This suggests that subjects reciprocally contribute to the returns payouts of those who contribute to them, which partially supports Result 7. In the fixed effects model, “No. Member” positively affects *α*_1_ at the five percent significance level. Both models suggest that the order of payout receipt does not significantly affect *α*_1_, which is consistent with Result 9.

## Conclusions

We conducted a laboratory experiment to show a mechanism for solving the default problem in a fixed ROSCA whose participants are shuffled randomly for each ROSCA cycle. We observed that the exclusion of defaulters through voting increased contribution rates before and after receiving returns payouts. We also found that a punishment rule, corresponding to that of Sugden [37] and the forfeiture rule of Koike et al. [20], did not improve contributions: without punishment rule, the subject voluntarily revenge others who had not contributed the subject (see Table 3). It is because all subjects know who receives the fund when in the fixed ROSCA. On the other hand, the punishment rule hinders voluntary revenge (see Table 3) and the external punishment works instead. As a result, there is no difference in contribution between Treatment P and Treatment B. Consequently, contrary to our theoretical predictions, the contribution rates and mean payoff of the subjects in Treatment V were significantly higher than in the other treatments. These results support those of field studies of ROSCAs, in which social sanctions through exclusion suppressed the problem of defaults even without external enforcement [22, 31].

Previous studies using the public goods game have shown that exclusion [40–42] and other endogenous group-formation [49, 55–57] promoted contribution level. The present investigation also supported those results even though our game structure differed from the public goods game (Table 6). However, an interesting finding in our ROSCA game was the observed voluntary direct reciprocity and voluntary retaliation (Treatment B and V). These two phenomena are not observed in the public goods game, where all the players contribute to the fund, which is distributed equally among all players. Another interesting finding of the present study was that, although we introduced an explicit costless punishment rule, it did not improve the contribution rate (Treatments P and VP). In our experiment, we could not clarify the relationship between "voluntary direct reciprocity and retaliation" and "explicit punishment".

**Table 6:** Comparison with the public goods experiments with exclusion.

	Rosca experiment	Public goods experiments with exclusion	
	Treatment V	Cinyabuguma et al. (2005)	Maier-Rigaud et al. (2010)
**Game stricture**	Players decide whether to contribute a fixed sum to a fund or not.	Players choose their contribution level to a public good.	
	Each player takes turns to receive the return.	Players share the total benefit from the public good.	
**Exclusion system**	Majority voting.	Majority voting.	
	Excluded players can join the group if they are not excluded in the next round.	Excluded players cannot join the group once they are excluded.	
**Result**	Contribution rate increases before and after each player receives the fund.	Contribution levels increase.	
**What promotes the contribution rate or level**			
Expelling low contributors?	Yes	Yes	
Reciprocity and retaliation?	Yes	No	
Threatening?	Yes	Yes	

Voluntary direct reciprocity and retaliation are reminiscent of the repeated game between two players, in which a player can behave either reciprocally or revengefully toward their opponent. However, the ROSCA game differs from the repeated game: the order of payout receipt influences reciprocity and retaliation in the ROSCA game. Further investigation of the ROSCA game should offer us new perspectives on the repeated game between two players.

Hechter [58] examined ROSCAs and argued that members can easily suppress non-contribution before receiving their payouts. This is because if a member fails to contribute to the fund before receiving their payout, the other members can prevent them from obtaining any funds. However, in our treatments, the subjects often failed to make a contribution before receiving their payout despite the punishment rule prohibiting the receipt of payouts by such non-contributors. This result reinforces the importance of exclusion based on reputation.

Exclusion based on majority voting has two characteristics: (1) low contributors are excluded from groups; and (2) low contributors who have been excluded sometimes have a chance to participate in a group again. Item (1) accords with the results of public good experiments [40, 41], but no experimental studies on exclusion have examined item (2). Sugden [37] proposed a mutual-aid game based on informal health insurance in England, with three elements: insurance members pay a subscription, one randomly chosen member receives the insurance money in each period, and a member previously awarded a payout remains eligible for future payouts. The mutual-aid game differs from ROSCAs in that every member has an equal chance of receiving the returns payout in each period. Sugden’s theoretical analysis showed that a kind of tit-for-tat strategy, which has the three features of “brave reciprocity,” “punishment,” and “reparation,” produces a stable equilibrium. Our results correspond to Sugden’s tit-for-tat strategy: in our experiment, the subjects behaved reciprocally and punished defectors by excluding them from the group. Thus, we consider that allowing excluded members to rejoin a group gives them a chance to make reparations for past defaults.

In this study, we investigated how social exclusion based on peer selection can prevent the problem of default in the fixed ROSCA. Besides fixed ROSCA, random and bidding ROSCAs are generally observed in the rural areas of developing countries. Future research is needed to examine the effectiveness of such social exclusion in the random and bidding ROSCAs and so verify the robustness of our results. In the random ROSCA, the subjects cannot use a retaliation strategy because the payout recipient is chosen randomly after subjects decide whether to contribute. Therefore the punishment rule, which did not work in the fixed ROSCA in our experiment, may be needed to sustain the random ROSCA. Because our study did not assume the existence of a costly punishment, it would be interesting to investigate how a costly punishment such as confiscating the property of the defaulter influences the sustainability of ROSCAs, even in a laboratory setting.

Further investigations in the fixed ROSCA are also needed to verify the robustness of our results. For instance, we will investigate how the knowledge that the subjects know in advance the game will be over at the 10th round affect the outcomes and how what points players start off with will change their behaviors. We will also examine if we can obtain the same results as our experiment when subjects are chosen from local people who often join real-world ROSCAs.

## Supporting information

S1 Appendix Subgame-perfect equilibrium for the ROSCA games experiment. (DOCX)

S1 Instruction Instructions for Treatment B. (DOCX)

S2 Instruction Instructions for Treatment P. (DOCX)

S3 Instruction Instructions for Treatment V. (DOCX)

S4 Instruction Instructions for Treatment VP. (DOCX)
