# Supplementary material for: Reciprocity and exclusion in informal financial institutions: An experimental study of rotating savings and credit associations
Source: PLoS One. 2018 Aug 29;13(8):e0202878. doi: 10.1371/journal.pone.0202878 (PMC6114866; doi:10.1371/journal.pone.0202878)
Supplement: S1 Appendix — (DOCX) [file pone.0202878.s001.docx]

**Supporting information**

Shimpei Koike, Mayuko Nakamaru, Tokinao Otaka, Hajime Shimao,

Ken-Ichi Shimomura, Takehiko Yamato

S1 Appendix: Subgame-Perfect Equilibrium for the ROSCA Games Experiment

We conducted experiments for four different versions of sequences of four-period ROSCA games. We formally prove that for all of the versions with which we experimented, the subgame-perfect equilibrium is that in each period, every player adopts the strategy of “pay no contribution regardless of history.”

We explain ten rounds of four-person four-period ROSCA games. Without loss of generality, assume that player $i$, $i\in\{1,2,3,4\}$, receives contributions from the other three players at period $i$. Then, one round consisting of a four-stage game is described as follows:

- Player 1 receives money simultaneously from players 2, 3, and 4 in period 1;
- Player 2 receives money simultaneously from players 1, 3, and 4 in period 2;
- Player 3 receives money simultaneously from players 1, 2, and 4 in period 3; and
- Player 4 receives money simultaneously from players 1, 2, and 3 in period 4.

A single stage game and a round comprising a four-stage game involve complete information, namely the possible actions and payoffs of players and the rules of the game are common knowledge among the players. In each period, every player is informed of the outcome of the previous period. At the beginning of each round, sets of players are shuffled. We thus assume that each player recognizes a round comprising a four-stage game as a new game sequence.

**Proposition B:** The subgame-perfect equilibrium for Treatment B (a four-person four-period ROSCA game) is that no player pays any money in any period.

**Proof.** We solve the subgame-perfect equilibrium for this game by backward induction.

Period 4: Period 4 is the final stage. In this stage, players 1, 2, and 3 simply lose some of their final payoffs if they contribute, so they have no incentive to do so. Hence, player 4 receives nothing.

Period 3: Players 1 and 2 lose some of their final payoffs by making contributions in period 3 and suffer no penalties in period 4 should they fail to contribute in period 3. In this stage, players 1 and 2 thus have no incentive to contribute. Hence, player 4 receives nothing in period 4 regardless of his contributions in period 3, and so he also has no incentive to pay. Therefore, player 3 receives nothing.

Period 2: Player 1 loses his payoff by making contributions in period 2 but suffers no penalties in periods 3 or 4 even if he does not contribute in period 2. This means player 1 has no incentive to make a contribution. Players 3 and 4 get nothing when their turns as recipients arrive, regardless of their contributions in period 2, and so have no incentive to make any contributions. Hence, player 2 receives nothing.

Period 1: Players 2, 3 and 4 receive nothing when it is their turn to receive a payout regardless of their contributions in period 2, and so have no incentive to contribute. Hence, player 1 receives nothing.

Thus, the subgame-perfect equilibrium for Treatment B is shown to be that every player adopts the strategy “pay no contribution to another player regardless of history.” Q.E.D.

We now consider a four-period ROSCA game that incorporates the following *punishment rule*: Suppose that a player does not contribute in some period before the arrival of his turn as payout recipient. Such failure to contribute makes the player ineligible to receive his payout and so one game in the sequence is skipped. At the beginning of each round, sets of players are shuffled as in Treatment B. We also assume that each player recognizes a round comprising a four-stage game as a new sequence of games.

**Proposition P:** The subgame-perfect equilibrium for Treatment P (a four-person four-period ROSCA game under the punishment rule) is that no player pays anything in any period.

**Proof.** We solve the subgame-perfect equilibrium for this game by backward induction.

Period 4: This period is the final stage. Because players 1, 2, and 3 simply lose portions of their payoffs if they contribute, they have no incentive to do so and therefore player 4 receives nothing.

Period 3: Player 4 knows that he will get nothing in period 4 regardless of his contributions in period 3, and so he has no incentive to contribute. Consequently, player 4 contributes nothing in period 3. Because of the *punishment rule*, no game is played in period 4. Players 1 and 2 lose part of their payoffs by making contributions in period 3 and lose nothing in period 3, when no game is played, and so they have no incentive to contribute. Hence, player 3 receives nothing.

Period 2: Suppose that the game is played in period. Player 4 knows that no game will be played in period 4, and therefore has no incentive to contribute. Player 3 knows that he will receive nothing in period 3 regardless of his contributions in period 2. Therefore he contributes nothing in period 2. Because of the *punishment rule*, no game is played in period 3. Player 1 loses part of their payoff by contributing in period 2 and loses nothing in period 3, when no game is played, and consequently he has no incentive to contribute. Hence, player 2 receives nothing.

Period 1: Player 4 has no incentive to contribute because he knows that no game will be played in period 4. Therefore, player 4 contributes nothing in period 1. Player 3 has no incentive to contribute because he knows that no game will be played in period 3. Therefore, player 3 contributes nothing in period 1. Player 2 has no incentive to contribute because he knows that he will get nothing in period 2 regardless of his contributions in the same period. Hence, player 2 contributes nothing in period 2. Because of the *punishment rule*, no game is played in period 2. Hence, player 1 receives nothing. Thus, the game is played only in period 1.

Therefore the subgame-perfect equilibrium for Treatment P is shown to be that every player adopts the strategy “pay no contribution to another player regardless of history” whenever the stage game is played. Q.E.D.

We next consider a “ten times longer” sequence of four-period ROSCA games with the following voting system: Round 1 is played in the same way as Treatment B. After round 1, the players are shuffled to form five new groups, each with four members. Within each group, the history of the members’ contributions in round 1 is public information. The members then vote to decide whom to exclude from round 2 (each member can choose as many players as he wants). Players chosen for exclusion by at least two members are barred from participation. (If each member is chosen for exclusion by two players, then all four players will be excluded so the group will play no game in round 2. If only one member is not excluded, then he becomes the only eligible player and so once again the group will play no game in round 2.) Then, round 2 is played by eligible members. After the completion of round 2, the 20 participants are reshuffled to form five new groups, each with four members. In each group, the history of the members’ contributions in rounds 1 and 2 is public information. The members then vote to decide whom to exclude from round 3. Players selected for exclusion by at least two members cannot participate in the games, and round 3 is played only by eligible members. After the completion of round 3, the process is repeated seven more times. At each period in round 1, every player chooses whether to contribute taking into account the history to that point. Conversely, in each of rounds 2–10, every player takes history into account and chooses whom to vote against and whether to contribute.

At the beginning of each round, sets of players are shuffled as in Treatments B and P. Players select strategies depending on history. We thus assume that each player recognizes ten rounds of four-stage games as a whole game.

**Proposition V:** The subgame-perfect equilibrium for Treatment V (ten rounds of four-person four-period ROSCA games under the exclusion by voting rule) is:

In round 1, regardless of period, every player pays nothing; and

In rounds 2–10, every player is “indifferent to voting” and pays nothing in any period.

**Proof.** We solve the subgame-perfect equilibrium for this game by backward induction. Consider round 10 of the sequence of four-period games, which is the final round.

Period 4 of round 10: Suppose that player 4 is not excluded. Period 4 is the final stage. If other players participate in this period, they have no incentive to contribute because to do so would decrease their final payoffs. If there are no other participants, then naturally there are no contributions. Either way, player 4 receives nothing. Meanwhile, if player 4 is excluded, then again he receives nothing.

Period 3 of round 10: Suppose that player 3 is not excluded. Player 4 knows that he will receive nothing in period 4 because the whole game is played with complete information. Consequently, player 4 contributes nothing in period 3 regardless of his participation in this period. Players 1 and 2 have no incentive to pay because they lose part of their payoffs by making contributions in period 3 and lose nothing in period 4 for not having contributed. Thus, players 1 and 2 contribute nothing in period 3 regardless of whether they participate in this period, with the result that player 3 receives nothing. If player 3 is excluded then naturally they also receive nothing.

Period 2 of round 10: Suppose that player 2 is not excluded. In this situation, players 3 and 4 know that they will receive nothing in periods 3 and 4, respectively, because the game is played with complete information. Therefore, neither player contributes in period 2 regardless of whether they participate. Player 1 has no incentive to contribute because by doing so he loses a payoff equal to his contribution at period 2 and faces no costly penalty for failure to contribute. Thus, player 1 contributes nothing in period 2 regardless of whether he participates, and consequently player 2 receives nothing. Meanwhile, if player 2 is excluded then once again they receive nothing.

Period 1 of round 10: Suppose that player 1 is not excluded in period 1. Players 2, 3 and 4 know that they will receive nothing in periods 2, 3 and 4, respectively, because the game is played with complete information. Therefore, players 2, 3 and 4 do not contribute anything in period 1 regardless of whether they participate in this period. Hence, player 1 receives nothing. Meanwhile, if player 1 is excluded, then once again they receive nothing.

Thus, we see by backward induction that every player adopts the strategy “pay no contribution to another player regardless of history” whenever the stage game is played.

Beginning of round 10: Players 1, 2, 3 and 4 know that they will get nothing in round 10. Therefore it does not matter for any player who participates in round 10 and so every player is “indifferent to voting” because his vote will not affect his payoff. Thus, for player $i$, the strategies “vote against player $j$” and “do not vote against player $j$”,$j\neq i$, (or a strategy of randomly selecting between the options if such a mixed strategy is permitted) are the best responses regardless of the history before round 10.

The above argument is true of not only round 10 but also of rounds 2–9. Hence, in rounds 2–10, every player is “indifferent to voting” and pays no money in any period.

The argument for round 1 is identical to that for Treatment B. Hence, in round 1, every player contributes nothing in every period. Q.E.D.

We finally consider a sequence of four-period ROSCA games under both the rule of exclusion by voting and the punishment rule: Round 1 is played as for Treatment B. After round 1 ends, the game participants are shuffled to form new five groups. In each group, the members vote to decide whom to exclude from round 2 as for Treatment V. Round 2 is played by the non-excluded members, and the punishment rule is applied as for Treatment P. Namely, a player is ineligible to receive funds in this round if he does not contribute in some period before his turn as recipient of the payout. After round 2 ends, the same process is repeated eight more times.

At each period in round 1, every player chooses whether to contribute, taking into account past history. Conversely, in each of rounds 2–10, every player chooses both whom to vote against and whether to contribute, taking into account history to that point. At the beginning of each round, sets of players are shuffled as in Treatments B, P and V. Players select strategies depending upon the history up to the current round and period. We thus assume that each player recognizes ten rounds of four-stage games as a whole game.

**Proposition VP:** The subgame-perfect equilibrium for Treatment VP (ten rounds of four-person four-period ROSCA games under both the rule of exclusion by voting and the punishment rule) is:

In round 1, every player pays no money in any period; and

In rounds 2–10, every player is “indifferent to voting” and pays no money in any period.

This proposition can be shown by combining the proofs of Propositions V and P.
