# Supplementary material for: Reciprocity and exclusion in informal financial institutions: An experimental study of rotating savings and credit associations
Source: PLoS One. 2018 Aug 29;13(8):e0202878. doi: 10.1371/journal.pone.0202878 (PMC6114866; doi:10.1371/journal.pone.0202878)
Supplement: S2 Instruction — (DOCX) [file pone.0202878.s003.docx]

**Supporting information**

Shimpei Koike, Mayuko Nakamaru, Tokinao Otaka, Hajime Shimao,

Ken-Ichi Shimomura, Takehiko Yamato

S2 Instruction: Instructions for Treatment P

Overview of the Experiment

The experiment comprises 10 rounds, each of which consists of four periods. Each group has four subjects. In the experiment, 100 tokens will be given to you in each period. You will not receive physical tokens, but please imagine that you have 100 tokens. Then please decide whether to “give” or “not give” 100 tokens to the collective fund of your group. One of the group members will receive the collective fund as a payout, and the fund will grow as the sequence of periods advances. The earlier you receive a payout and the more members contribute tokens, the larger the payoff you will obtain.

Your earnings will be determined according to your payoff in the experiment. As your payoff increases, so too will your earnings. Your payoff changes depending both on your own choices and those of other members.

Experiment Procedure

Step 1

Twenty subjects are randomly organized into five groups, each consisting of four subjects. You belong to one of these groups. You will not be able to identify the other members of your group. Similarly, other members of your group will be unaware whether you belong to the group.

Step 2

The computer randomly determines the “order payout receipt”. The order will be shown on your computer screen. Please check your position in the order.

Example 1:

If you are the second recipient, the following information will show on your computer screen:

Step 3

Each round consists of four periods. Each period proceeds as follows.

At the beginning of the period you receive 100 tokens. Your position in the order of payout receipt determines what you do in each of the four periods.

(a) In the periods when you are not scheduled to receive the payout:

You can choose either to “**give”** 100 tokens to the collective fund or to **“not give”**.

If you choose “GIVE”, then all 100 tokens will be added to the fund, and you will lose 100 tokens. You will not save anything and your payoff will be 0 in this period.

If you choose “NOT GIVE”, then you will save 100 tokens and your payoff in this period will be 100. However, should you not yet have received a payout, by choosing “NOT GIVE” you will lose your right to receive a payout in future. In this case, on the arrival of your turn as the payout recipient, the other group members then will not need to choose “GIVE” or “NOT GIVE” and all members will simply save 100 tokens.

(b) In the period when you are scheduled to receive the payout:

If you have already chosen “NOT GIVE” at least once, you cannot receive the payout. In this case, all members save 100 tokens and the period ends.

If you have never chosen “NOT GIVE”, you can receive the payout funded by the other members of your group. You do not need to choose “GIVE” or “NOT GIVE”. The size of your payout depends on how many other members of your group give 100 tokens in this period.

Your payoff in one round consisting of four periods is determined by both “the order of payout receipt” and “the number of other members of your group who give 100 tokens to the fund” as follows:

|  |  | The number of members who choose "GIVE" | | | |
| --- | --- | --- | --- | --- | --- |
|  |  | 0 | 1 | 2 | 3 |
| The order of payout receipt | First period | 100 | 320 | 539 | 759 |
|  | Second period | 100 | 269 | 438 | 607 |
|  | Third period | 100 | 230 | 360 | 490 |
|  | Fourth period | 100 | 200 | 300 | 400 |

More specifically, your payoff in a round is calculated as follows:

**Your payoff =**

**(the number of members who have invested in the collective fund) * 100 * 1.3^(4 − your receiving order)^ +100**

This means that in each period after you receive the payout its value is multiplied by 1.3. Therefore, the earlier you receive the payout, or the more members give 100 tokens to fund your payout, the larger a payoff you can obtain. Meanwhile, “+ 100” in the second term on the right hand side means that you can automatically save 100 tokens that are given to you at the beginning of the period in which you receive the payout.

After all members finish making decisions in each period, two tables are displayed on your screen.

Example: Result and Payoff Tables after the second period.

The table on the left indicates **who gave 100 tokens and who did not**. The mark “○” indicates a choice to “GIVE”, “x” means a choice to “NOT GIVE”, and “-” means you made no decision because you were the scheduled recipient in that period. The table on the right indicates **the payoffs of all members in each period**.

This process is repeated four times until every member has a chance to receive the payout. At this point the round is concluded.

Example:

Taking a specific example, we will show you how the experiment proceeds. Suppose that the order of payout receipt is

A1 → B1 (you) → C1 → D1.

The first period:

You have 100 tokens.

Player A1 receives the payout in this period.

You can choose either “GIVE” or “NOT GIVE”.

Suppose that you choose “GIVE”.

Then your payoff in this period will be **0**.

Also, suppose that C1 and D1 choose “GIVE”.

The choices and payoffs of each member will be displayed on your screen as follows:

A1 receives 300 tokens and A1's payout will be **759 at the end of** the fourth period:

300 * 1.3^(4−1)^ + 100 ≒ 759.

The second period:

You have 100 tokens once again.

In this period, you are the scheduled recipient of the payout and do not need to choose anything.

The size of the payout is determined by the choices of the other members.

Suppose that A1 and C1 choose “GIVE”, and D1 chooses “NOT GIVE”.

D1 loses the right to receive the payout in the fourth period because he/she chose “NOT GIVE” in this period. All members will thus save 100 tokens in the fourth period, and will not need to make any choices.

The current and past choices and payoffs of each member will be displayed on your screen as follows:

You receive 200 tokens and your payoff will be **438 at the end of** the fourth period:

　200 * 1.3^(4−2)^ + 100 = 438.

The third period:

You have 100 tokens once again.

C1 receives the payout. You can choose either “GIVE” or “NOT GIVE”.

Suppose that you choose “NOT GIVE”.

Then your payoff in this period will be **100**.

Suppose that A1 and D1 choose “NOT GIVE”.

The current and past choices and payoffs of each member will be displayed on your screen as follows:

C1 receives 0 tokens and his or her payoff will be **100 at the end of** the fourth period:

0 * 1.3^(4−3)^ + 100 = 100.

The fourth period:

You have 100 tokens once again.

D1 is the scheduled recipient of the payout, but because he/she chose “NOT GIVE” in the second period, he/she lost the right to receive the payout. Therefore, you do not need to choose “GIVE” or “NOT GIVE”. You can automatically save 100 tokens, and your payoff in this period is **100**.

The current and past choices and payoffs of each member will be displayed on your screen as follows:

This is the end of the round. The payoff of each participant in this round is the sum of his/her payoffs over all four periods, as the following table illustrates:

|  | A1 | B1 (You) | C1 | D1 |
| --- | --- | --- | --- | --- |
| Period 1 | 759 | 0 | 0 | 0 |
| Period 2 | 0 | 438 | 0 | 100 |
| Period 3 | 100 | 100 | 100 | 100 |
| Period 4 | 100 | 100 | 100 | 100 |
| Sum | 959 | 638 | 200 | 300 |

Step 4

Go back to Step 1. In the next round, the 20 subjects are again randomly organized into five new groups. The same procedure will be repeated. As group members are randomly chosen, your group in the second round may or may not contain members from your group in the first round.

Your choices in the first round do not affect your right to receive the payout in the second round. At the beginning of the second round, you have the right to receive the payout even if you chose “NOT GIVE” in the first round. Of course, if you choose “NOT GIVE” in the second round, you will lose the right to receive the payout in that round.

This procedure will be repeated **10 times**. The experiment consists of 10 rounds, each comprising four periods.

After choosing either “GIVE” or “NOT GIVE”, please write down the reasons for your decision in your record sheet.

Earnings

You can calculate your earnings, to be paid by us, as follows.

**Your earnings = (the sum of your payoffs over 10 rounds) × 0.75 (JPY).**

Any decimal places are rounded up.

This is the end of the instructions. Please raise your hand if you have any questions.

Let us start the experiment. First, please take 3 minutes to read the instructions to make sure you understand the rules of the experiment completely.

Please raise your hand silently if you have any questions. The experimenters will come to you. Do not communicate with the other subjects. Thank you.
