# Supplementary material for: Reciprocity and exclusion in informal financial institutions: An experimental study of rotating savings and credit associations
Source: PLoS One. 2018 Aug 29;13(8):e0202878. doi: 10.1371/journal.pone.0202878 (PMC6114866; doi:10.1371/journal.pone.0202878)
Supplement: S4 Instruction — (DOCX) [file pone.0202878.s005.docx]

**Supporting information**

Shimpei Koike, Mayuko Nakamaru, Tokinao Otaka, Hajime Shimao,

Ken-Ichi Shimomura, Takehiko Yamato

S4 Instruction: Instructions for Treatment VP

Overview of the Experiment

The experiment comprises 10 rounds, each of which consists of four periods. Each group has four subjects. In the experiment, 100 tokens will be given to you in each period. You will not receive physical tokens, but please imagine that you have 100 tokens. Then please decide whether “to give” or “not give” 100 tokens to the collective fund of your group. One of the group members will receive the collective fund as a payout, and the collective fund will grow as the sequence of periods advances. The earlier you receive a payout and the more members contribute tokens, the larger the payoff you will obtain.

At the beginning of each round, you will vote to decide who should be excluded from the group. The voting system will be explained later.

Your earnings will be determined according to your payoff in the experiment. As your payoff increases, so too will your earnings. Your payoff changes depending both on your own choices and those of other members.

Experiment Procedure

Step 1

Twenty subjects are randomly organized into five groups, each consisting of four subjects. You belong to one of these groups. You will not be able to identify the other members of your group. Similarly, other members of your group will be unaware whether you belong to the group.

Step 2

The computer randomly determines the “order of payout receipt”. The order will be shown on your computer screen. Please check your position in the order.

Example 1:

If you are the second recipient, the following information will show on your computer screen:

Step 3

Each round consists of four periods. Each period proceeds as follows.

At the beginning of the period you receive 100 tokens. Your position in the order of payout receipt determines what you do in each of the four periods.

(a) In the period when you are not scheduled to receive the payout:

You can choose either to **“give”** 100 tokens to the collective fund or to **“not give”**.

If you choose “GIVE”, then all 100 tokens will be added to the fund, and you will lose 100 tokens. You will not save anything and your payoff will be 0 in this period.

If you choose “NOT GIVE”, then you will save 100 tokens and your payoff in this period will be 100.

However, should you not yet have received a payout, by choosing “NOT GIVE” you will lose your right to receive a payout in future. On the arrival of your turn as the payout recipient, the other group members will not need to choose “GIVE” or “NOT GIVE” and all members will simply save 100 tokens.

(b) In the period when you are scheduled to receive the payout:

If you have already chosen “NOT GIVE” at least once, you cannot receive the payout. In this case, all members save 100 tokens and the period ends.

If you have never chosen “NOT GIVE”, you can receive the payment founded by the other members of your group. You do not need to choose “GIVE” or “NOT GIVE”. The size of your payout depends on how many other members of your group give 100 tokens in this period.

Your payoff in one round consisting of four periods is determined by both “the order of payout receipt” and “the number of other members of your group who give 100 tokens to the fund” as follows:

|  |  | The number of members who choose "GIVE" | | | |
| --- | --- | --- | --- | --- | --- |
|  |  | 0 | 1 | 2 | 3 |
| The order of payout receipt | First period | 100 | 320 | 539 | 759 |
|  | Second period | 100 | 269 | 438 | 607 |
|  | Third period | 100 | 230 | 360 | 490 |
|  | Fourth period | 100 | 200 | 300 | 400 |

More specifically, your payoff in a round is calculated as follows:

**Your payoff =**

**(the number of members who have invested in the collective fund) * 100 * 1.3^(4 − your receiving order)^ +100**

This means that in each period after you receive the payout its value is multiplied by 1.3. Therefore, the earlier you receive the payout, or the more members give 100 tokens to fund your payout, the larger a payoff you can obtain. Meanwhile, “+ 100” in the second term on the right hand side means that you can automatically save 100 tokens that are given to you at the beginning of the period in which you receive the payout.

After all members finish making decisions in each period, two tables are displayed on your screen.

Example: Result and Payoff Tables after the second period.

The table on the left indicates **who gave 100 tokens and who did not**. The mark “○” indicates a choice to “GIVE”, “x” means a choice to “NOT GIVE”, and “-“ means you made no decision because you were the scheduled recipient in that period. The table on the right indicates **the payoffs of all members in each period**.

This process is repeated four times until every member has a chance to receive the payout. At this point the round is concluded.

Example:

Taking a specific example, we will show you how the experiment proceeds. Suppose that the order of payout receipt is

A1 → B1 (you) → C1 → D1.

The first period:

You have 100 tokens.

Player A1 receives the payout in this period.

You can choose either “GIVE” or “NOT GIVE”.

Suppose that you choose “GIVE”.

Then your payout in this period will be **0**.

Also, suppose that C1 and D1 choose “GIVE”.

The choices and payouts of each member will be displayed on your screen as follows:

A1 receives 300 tokens and A1’s payout will be **759 at the end of** the fourth period:

300 * 1.3^(4−1)^ + 100 ≒ 759.

The second period:

You have 100 tokens once again.

In this period, you are the scheduled recipient of the payout and do not need to choose anything.

The payout is determined by the choices of the other members.

Suppose that A1 and C1 choose “Give”, and D1 chooses “Not Give”.

D1 loses the right to receive a payout in the fourth period because he/she chose “NOT GIVE” in this period. All members thus will save 100 tokens in the fourth period, and will not need to make any choices.

The current and past choices and payouts of each member will be displayed on your screen as follows:

You receive 200 tokens and your payoff will be **438 at the end of** the fourth period:

　200 * 1.3^(4−2)^ + 100 = 438.

The third period:

You have 100 tokens once again.

C1 is the scheduled recipient of the payout. You can choose either “GIVE” or “NOT GIVE”.

Suppose that you choose “NOT GIVE”.

Then your payoff in this period will be **100**.

Suppose that A1 and D1 choose “NOT GIVE”.

The current and past choices and payoffs of each member will be displayed on your screen as follows:

C1 receives 0 tokens and his or her payoff will be **100 at the end of** the fourth period:

0 * 1.3^(4−3)^ + 100 = 100.

The fourth period:

You have 100 tokens once again.

D1 is the scheduled recipient of the payout, but he/she chose "NOT GIVE" in the second period, and so lost the right to receive any payout. Therefore, you do not need to choose “GIVE” or “NOT GIVE”. You can automatically save 100 tokens, and your payoff in this period is **100**.

The current and past choices and payoffs of each member will be displayed on your screen as follows:

This is the end of the round. The payoff of each participant in this round is the sum of his/her payoffs over four periods, as the following table illustrates:

|  | A1 | B1 (You) | C1 | D1 |
| --- | --- | --- | --- | --- |
| Period 1 | 759 | 0 | 0 | 0 |
| Period 2 | 0 | 438 | 0 | 100 |
| Period 3 | 100 | 100 | 100 | 100 |
| Period 4 | 100 | 100 | 100 | 100 |
| Sum | 959 | 638 | 200 | 300 |

Step 4

Go back to Step 1. In the next round, the 20 subjects are randomly organized into five new groups. The same procedure will be repeated. As group members are randomly chosen, your group in the second round may or may not contain members from your group in the first round.

Your choices in the first round do not affect your right to receive a payout in the second round. At the beginning of the second round, you have the right to receive a payout even if you chose “NOT GIVE” in the first round. Of course, if you choose “NOT GIVE” in the second round, you will lose the right to receive a payout in that round.

Step 5

Starting in the second round, you need to decide who should be excluded from the group by vote. The voting process decides eligible participants in each round.

The following information about candidates for membership of the second round is shown on your computer screen. The second column of the table indicates **how many contribution decisions each candidate made in the previous round**, and the third column indicates **how many times each candidate actually contributed**.

Based on this information, please vote on whether each candidate should be excluded from the group. Mark candidates you wish to expel with “x”, and mark those you do not wish to expel with “○”. Votes for or against yourself are not counted but you must still choose “x” or “○”.

Candidates who win more than two “○” votes can participate in this round, and candidates who receive more than two “x” votes cannot participate. If you are expelled from the group, you will receive 100 tokens in each period, meaning your payoff in this round will be **400**.

Example:

Taking a specific example, suppose that a voting result is as follows.

The second row of the table indicates the number of “○” votes and the third row indicates whether you can participate in the group.

In this case, Candidate 2 is excluded from the group and the other candidates are allowed to participate. Candidate 2 receives 100 tokens in each period and his/her payoff in the second round is 400.

The members of the group carry out the following process even if they number less than four.

The computer randomly determines “the order of payout receipt” for the members.

You get 100 tokens in each of the four periods.

You will decide whether “to give” or “not give” 100 tokens as many times as the group has members, and then, at the end of the fourth period, your payoff is as follows.

In the case where the group has three members:

|  |  | The number of members who choose “GIVE” | | |
| --- | --- | --- | --- | --- |
|  |  | 0 | 1 | 2 |
| The order of payout receipt | First period | 100 | 269 | 438 |
|  | Second period | 100 | 230 | 360 |
|  | Third period | 100 | 200 | 300 |

**Your payoff =**

**(the number of members who have invested in the communal fund) * 100 * 1.3^(3 − your position in the order of payout receipt)^ +100**

In the case where the group has two members:

|  |  | The number of members who choose “GIVE” | |
| --- | --- | --- | --- |
|  |  | 0 | 1 |
| The order of payout receipt | First period | 100 | 230 |
|  | Second period | 100 | 200 |

**Your payoff =**

**(the number of members who have invested in the communal fund) * 100 * 1.3^(2 − your position in the order of payout receipt)^ +100**

You automatically save 100 tokens in the rest of the period, and then the round is over.

Example:

Besides the last example, suppose that Candidate 1, Candidate 4, and you are the group members.

The computer randomly determines “the order of payout receipt”, according to which you are A2 and the first recipient. B2 is the second recipient and C2 is the third recipient but **you cannot know which candidate is B2, and which candidate is C2.**

The first period:

You have 100 tokens.

In this period, you are the scheduled recipient of the payout and do not need to choose anything.

The payoff is determined by the choices of the other members.

Suppose that B2 chooses “NOT GIVE”, and C1 chooses “GIVE”.

The choices and payoffs of each member will be displayed on your screen as follows:

You receive 300 tokens and your payoff will be **269 at the end of** the fourth period:

100 * 1.3^(3−1)^ + 100 ≒ 269.

The second period:

You have 100 tokens once again.

B2 is the scheduled recipient of the payout, but he/she chose “NOT GIVE” in the first period, and so lost the right to receive any payment. Therefore, you do not need to choose “GIVE” or “NOT GIVE”. You can automatically save 100 tokens, and your payoff in this period is **100**.

The current and past choices and payoffs of each member will be displayed on your screen as follows:

The third period:

You have 100 tokens once again.

C2 receives the payout. You can choose either “GIVE” or “NOT GIVE”.

Suppose that you choose “NOT GIVE”.

Then your payoff in this period will be **100**.

Suppose that B2 chooses "GIVE".

The current and past choices and payoffs of each member will be displayed on your screen as follows:

C2 receives 100 units and his or her payoff is **200** **at the end of** the fourth period:

100 * 1.3^(3−3)^ +100 = 200.

The fourth period:

You have 100 tokens once again. You do not need to choose “GIVE” or “NOT GIVE”, because all members have already had a chance to receive a payout. Your payoff in this period is 100.

This is the end of the second round. The payoff of each participant in this round is the sum of his/her payoffs over four periods as the following table illustrates:

|  | A2(You) | B2 | C2 | Absentee |
| --- | --- | --- | --- | --- |
| Period 1 | 269 | 100 | 0 | 100 |
| Period 2 | 100 | 100 | 100 | 100 |
| Period 3 | 100 | 0 | 200 | 100 |
| Period 4 | 100 | 100 | 100 | 100 |
| Sum | 569 | 300 | 400 | 400 |

This procedure will be repeated until **the 10th round**.

The third round:

The 20 subjects are again randomly organized into five new groups. Please confirm the voting process again. The following information about candidates for membership of the third round is shown on your computer screen. The second column of the table indicates **how many contribution decisions each candidate made in previous rounds** and the third column of the table indicates **how many times each candidate actually contributed.**

In the following case, Candidate 1 could not participate in the previous group, and therefore the numbers of his or her contributions and contribution decisions have not changed since the last round. Meanwhile, your contribution decisions increased from the last round because you decided in the third period of the round.

After choosing either “GIVE” or “NOT GIVE”, please write down the reasons for your decision in your record sheet.

Earnings

You can calculate your earnings, to be paid by us, as follows.

**Your earnings = (the sum of your payoffs over 10 rounds) × 0.75 (JPY).**

Any decimal places are rounded up.

This is the end of the instructions. Please raise your hand if you have any questions.

Let us start the experiment. First, please take 3 minutes to read the instructions to make sure you understand the rules of the experiment completely.

Please raise your hand silently if you have any questions. The experimenters will come to you. Do not communicate with the other subjects. Thank you.
